# Suggested Cut-Off Values for Vitamin D as a Risk Marker for Total and Cardiac Death in Patients with Suspected Acute Coronary Syndrome

**DOI:** 10.3389/fcvm.2016.00004

**Published:** 2016-02-26

**Authors:** Patrycja A. Naesgaard, Ricardo A. León de la Fuente, Stein Tore Nilsen, Volker Pönitz, Trygve Brügger-Andersen, Heidi Grundt, Harry Staines, Dennis W. T. Nilsen

**Affiliations:** ^1^Department of Cardiology, Stavanger University Hospital, Stavanger, Norway; ^2^Department of Clinical Science, University of Bergen, Bergen, Norway; ^3^Cardiology Research Institute, Catholic University of Salta, Salta, Argentina; ^4^Department of Research, Stavanger University Hospital, Stavanger, Norway; ^5^Department of Clinical Medicine, University of Bergen, Bergen, Norway; ^6^Department of Internal Medicine, Stavanger University Hospital, Stavanger, Norway; ^7^Sigma Statistical Services, Balmullo, UK

**Keywords:** vitamin D, acute coronary syndrome, total mortality, cardiac death, cut-off levels, vitamin D deficiency

## Abstract

**Background:**

Several studies have demonstrated an association between low vitamin D levels and cardiovascular risk. Vitamin D cut-off levels are still under debate.

**Objectives:**

To assess two cut-off levels, 40 and 70 nmol/L, respectively, for vitamin D measured as 25-hydroxyvitamin D in chest pain patients with suspected acute coronary syndrome.

**Methods:**

We investigated 1853 patients from coastal-Norway and inland Northern-Argentina. A similar database was used for pooling of data. Two-year follow-up data including all-cause mortality, cardiac death, and sudden cardiac death in the total patient population were analyzed, applying univariate and multivariable analysis.

**Results:**

Two hundred fifty-five patients with known vitamin D concentrations died. In the multivariable analysis, there was a decrease in total mortality above a cut-off level of 40 nmol/L and a decrease in cardiac death above a cut-off level of 70 nmol/L [HRs of 0.66 (95% CI, 0.50–0.88), *p* = 0.004 and 0.46 (95% CI, 0.22–0.94), *p* = 0.034, respectively].

**Conclusion:**

Vitamin D cut-off levels of 40 and 70 nmol/L were related to total mortality and cardiac death, respectively.

## Introduction

Vitamin D is synthesized in the skin on exposure to UV light, or obtained from the diet, mainly from fatty fish (1). Vitamin D deficiency in humans is prevalent and cardiovascular disease (CVD) is the primary cause of mortality worldwide (2). Several observational and epidemiological studies have demonstrated an association between low levels of vitamin D and (CVD) and mortality (3–8).

We have previously studied separately two populations, one from coastal Norway and one from inland Northern-Argentina, with respect to the prognostic significance of vitamin D, measured as 25-hydroxyvitamin D [25(OH)D] in acute coronary syndrome (ACS). Fish is frequently consumed by the costal population of Norway, and is less preferred by the inland beef-consuming population in Northern Argentina. At the time of this study, vitamin D fortification in food had not been introduced in that country. Furthermore, the subtropical location of Salta, Argentina, is associated with a higher exposure to sun in comparison to that of the temperate location of Norway. Our two patient populations were divided into 25(OH)D quartiles, comparing patients with high and low levels of vitamin D. We found that vitamin D was an independent prognostic biomarker in relation to total mortality and cardiac death in the Argentinean population, whereas in the Norwegian population its prognostic utility was less evident, but with a clear trend supporting the findings obtained in the Argentinean study (9, 10).

Optimal and exact cut-off levels of vitamin D are still under debate (8, 11–13). Additionally, studies have shown that high levels of vitamin D are also associated with increased mortality (3, 14).

In the present study, we have pooled our two populations and grouped them according to gender, in order to better assess the prognostic significance of 25(OH)D according to (1) 25(OH)D groups and (2) two cut-off values. We have added information based on a healthy blood donor population from both countries in order to obtain a reference value for vitamin D.

## Materials and Methods

### Study Design and Patient Population

Participants in the present analysis were pooled from two studies, both designed to evaluate the prognostic utility of serum 25(OH)D status in patients with chest pain and ACS.

The RACS study (Risk Markers in the Acute Coronary Syndrome) (ClinicalTrials.gov NCT00521976) was a single-center prospective observational study in which 871 patients with chest pain and suspected ACS were consecutively admitted to the Stavanger University Hospital from November 2002 to October 2003.

The ARgentinean Risk Assessment Registry in the Acute Coronary Syndrome, the “ARRA-RACS Study” (Ref. ClinicalTrial.gov identifier: NCT01377402), was performed at nine hospitals in the Province of Salta, Northern Argentina, in which 982 patients with chest pain and suspected ACS were hospitalized from December 2005 to January 2009.

The details, including blood sampling procedures, laboratory measurements, and exclusion criteria of the RACS and ARRA-RACS studies, have been published previously (9, 10, 15).

The primary outcome in both studies was 2-year all-cause mortality, and the secondary outcomes included cardiac death and sudden cardiac death (SCD). The population was divided into three groups according to the following 25(OH)D: ≤40 nmol/L, between >40 and ≤70 nmol/L, and >70 nmol/L, and two cut-off levels; ≤40 nmol/L and ≤70 nmol/L. The prognostic impact within the suggested 25(OH)D levels was analyzed applying univariate and multivariable analysis.

The blood bank study included blood samples from 86 healthy individuals, with an equal number of males and females, recruited from the blood bank in Stavanger, Norway, and 104 healthy individuals (53 females and 49 males) recruited from two blood banks in Salta, Argentina. Their information was treated anonymously.

25(OH)D measurements were carried out at the Department of Medical Biochemistry at Stavanger University Hospital, as previously described (9).

## Statistical Analysis

Patients were divided into three categories according to their 25(OH)D levels. Results are presented for the complete 2-year follow-up period and after censoring the events in the first 3 months (91 days). Approximately, normally distributed variables were given as mean and standard deviation (SD) within vitamin D groups. The Chi-square test for association was applied between the 25(OH)D and categorical variables at baseline. The one-way ANOVA was used to test for the equality of means of scale variables (e.g., age) among vitamin D categories and the two-sample *t*-test was used for comparing means of two samples. Stepwise Cox multivariable proportional hazards regression models with total death, cardiac death, and SCD, as the dependent variable, and 25(OH)D categories and other variables as potential independent predictors (listed below) were fitted. To examine the differences in prognosis between subjects in the higher vs. the lowest 25(OH)D group, we adjusted for gender, age, smoking, hypertension, index diagnosis, diabetes mellitus, congestive heart failure (CHF) [defined by Killip–Kimball class (16) at admission; patients in classes 2–4 were classified as CHF patients and patients in class 1 were classified as non-CHF], history of previous coronary heart disease (i.e., history of either angina pectoris, MI, coronary artery bypass graft, or percutaneous coronary intervention), hypercholesterolemia/use of statins, TnT > 0.01 ng/mL, estimated glomerular filtration rate, high sensitivity C-reactive protein, B-type natriuretic peptide, body mass index (BMI) (kg/m^2^), month of sampling, country, and use of betablockers prior to enrollment. The hazard ratios (HRs) are presented with 95% confidence interval (CI). The Kaplan–Meier product limits were used for plotting time-to-event and the log-rank test was used to compare survival curves across 25(OH)D groups. The added prognostic value by including 25(OH)D levels to the significant confounders in the Cox regression model was assessed by using the log-likelihood test. The statistical analyses were performed using the statistical package SPSS version 19.0. All tests were two sided with a significance level of 5%. Highly significant differences were based on a two-tailed significance level of 1%.

## Ethics Statement

The RACS study was approved by the Regional Board of Research Ethics and the Norwegian Health Authorities and conducted in accordance with the Helsinki Declaration of 1971, as revised in 1983.

The ARRA-RACS study was approved by the Ethics Committee at the Board of Medical School of Salta and conducted in accordance with the Helsinki Declaration of 1971, as revised in 1983. At San Bernardo Hospital and Sanatorio El Carmen, the study was also approved by the local Ethics Committee and Institutional Review Board of San Bernardo Hospital and the Institutional Review Board of Sanatorio El Carmen, respectively. The Norwegian biobank containing Argentinean blood samples was approved by the Regional Board of Research Ethics and the Norwegian health authorities. This study was monitored by Stavanger Health Research, Stavanger, Norway.

The blood bank study was approved by the Regional Board of Research Ethics.

Written informed consent was obtained from all patients in all three studies.

## Results

A total of 1853 (60% males and 40% females) patients were included in the study; 982 from Argentina and 871 from Norway. Samples for 25(OH)D analysis were not available for 2 Argentinean and 10 Norwegian patients, and 1 implausibly high 25(OH)D value for a Norwegian patient was ignored. Four Argentinean patients with known 25(OH)D values were lost to follow-up and, hence, their mortality status is unknown. Thus, both survival and 25(OH)D values are known for 1836 (976 Argentinean and 860 Norwegian) patients.

Mean age (±SD) in the combined patient population was 65.7 ± 14.3 (males 63.0 ± 14.2 years and females 69.8 ± 13.5 years). Mean 25(OH)D levels were 51.9 ± 18.0 nmol/L in the total patient population, 54.1 ± 17.9 nmol/L in males and 48.4 ± 17.5 nmol/L in females. The baseline characteristics according to 25(OH)D concentrations at admission in the total patient population are shown in Table 1.

**Table 1:** **Baseline characteristics according to 25(OH)D concentrations at admission in the total patient population**.

	Concentrations of 25(OH)D			
Characteristics *n* (%)	≤40 nmol/L	40–70 nmol/L	>70 nmol/L	*p*-value
Mean 25(OH)D (nmol/L)^a^	31.6 ± 6.0	54.2 ± 8.1	82.4 ± 12.4	<0.001
Age, years^a^	68.3 ± 14.2	64.5 ± 14.4	64.9 ± 14.0	<0.001
Male, *n* (%)	255 (49.4)	677 (63.9)	180 (68.2)	<0.001
Smoking status, *n* (%)				0.50
Current smoker, *n* (%)	139 (27.3)	264 (25.2)	61 (23.3)	
Past smoker, *n* (%)	243 (47.6)	484 (46.1)	122 (46.7)	
Never smoked, *n* (%)	128 (25.1)	301(28.7)	78 (29.9)	
Angina pectoris, *n* (%)	161 (31.2)	349 (32.9)	88 (33.3)	0.75
CHF, *n* (%)				
Killip classes 2–4	156 (30.2)	189 (17.8)	52 (19.7)	<0.001
History of previous MI, *n* (%)	124 (24.0)	205 (19.3)	52 (19.7)	0.09
CABG, *n* (%)	38 (7.4)	76 (7.2)	20 (7.6)	0.97
PCI, *n* (%)	50 (9.7)	106 (10.0)	29 (11.0)	0.85
Hypertension, *n* (%)	296 (57.4)	553 (52.2)	145 (54.9)	0.14
History of DM 1, *n* (%)	9 (1.8)	12 (1.1)	2 (0.8)	0.43
History of DM 2, *n* (%)	117 (22.9)	155 (14.7)	26 (9.9)	<0.001
STEMI, *n* (%)	71 (13.9)	166 (15.8)	35 (13.3)	0.45
TnT release, *n* (%)	255 (49.4)	476 (44.9)	123 (46.6)	0.25
eGFR (μmol L^−1^)^a^	71.4 ± 29.3	74.4 ± 26.0	68.0 ± 23.8	0.001
Cholesterol/statin, *n* (%)	157 (30.4)	326 (30.8)	100 (37.9)	0.07
Beta-blocker, *n* (%)	157 (30.7)	323 (30.7)	81 (31.0)	0.99
Known CHD, *n* (%)	243 (47.4)	497 (47.0)	126 (47.9)	0.97
BMI (kg/m^2^)^a^	27.0 ± 5.0	27.1 ± 4.4	25.8 ± 3.9	<0.001
BNP quartiles				<0.001
Q1	111 (21.9)	270 (26.0)	72 (28.1)	
Q2	98 (19.3)	288 (27.7)	64 (25.0)	
Q3	138 (27.2)	252 (24.2)	61 (23.8)	
Q4	160 (31.6)	230 (22.1)	59 (23.0)	
hsCRP quartiles				0.009
Q1	105 (20.4)	281 (26.5)	76 (28.8)	
Q2	114 (22.2)	271 (25.6)	70 (26.5)	
Q3	144 (28.0)	256 (24.2)	61 (23.1)	
Q4	151 (29.4)	251 (23.7)	57 (21.6)	
Country				0.002
Argentina	284 (55.0)	582 (54.9)	114 (43.2)	
Norway	232 (45.0)	478 (45.1)	150 (56.8)	

*^a^Mean ± SD*.

*SD, standard deviation 25(OH)D, 25-hydroxyvitamin D; CHF, congestive heart failure; MI, myocardial infarction; CABG, coronary artery bypass grafting; PCI, percutaneous coronary intervention; DM, diabetes mellitus; STEMI, ST-elevation myocardial infarction; TnT, troponin T; eGFR, estimated glomerular filtration rate; CHD, coronary heart disease; BMI, body mass index; BNP, B-type natriuretic peptide; hsCRP, high sensitivity C-reactive protein*.

### Prognosis Estimates Related to Different Vitamin D Concentrations

After a 2-year follow-up, 255 patients with known 25(OH)D values had died. Of these, 150 deaths were caused by a cardiac event, of whom, 75 patients died suddenly.

**Figure 1:**
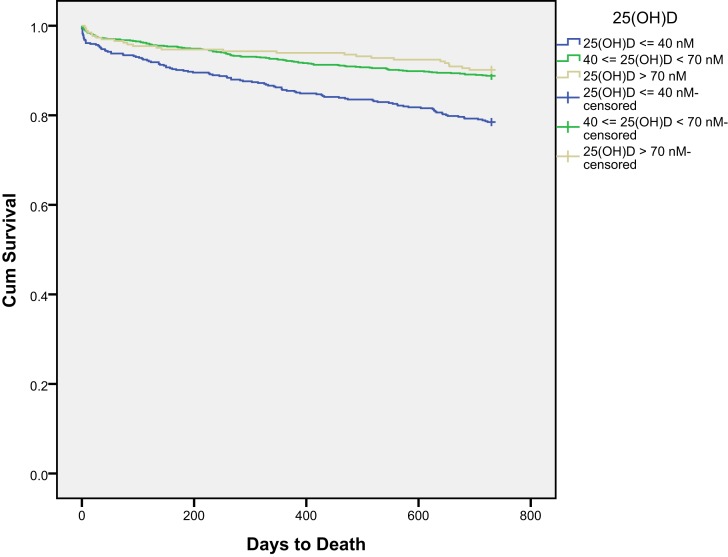
**Kaplan–Meier survival curves for all-cause mortality in the three 25(OH)D groups for all patients**.

**Figure 2:**
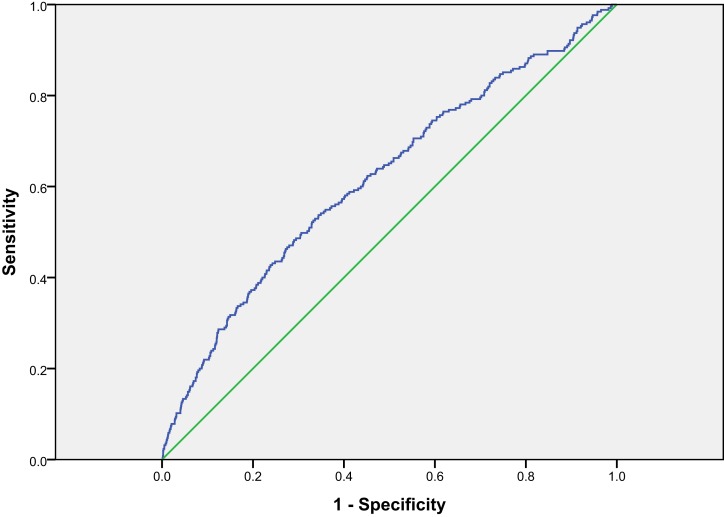
**Receiver-operated characteristics curve for 25(OH)D for all-cause mortality**.

Kaplan–Meier survival curves for all-cause mortality in three 25(OH)D groups are presented in Figure 1. Receiver-operated characteristics (ROC) curve for 25(OH)D for all-cause mortality is shown in Figure 2. The area under the ROC for 25(OH)D was 0.619 (*p* < 0.001). Using 40 nmol/L as threshold, the sensitivity was 0.435 and specificity was 0.744. Using 70 nmol/L as threshold, the sensitivity was 0.898 and specificity was 0.150.

In the univariate Cox regression analysis, there was a highly statistically significant difference between the upper (>70 nmol/L) and middle (40–≤70 nmol/L) as compared to the lowest group of 25(OH)D with respect to all-cause mortality, cardiac death, and SCD. These findings were maintained for total death in the multivariable Cox regression analysis, with HRs of 0.56 (95% CI, 0.34–0.92), *p* = 0.021 and 0.69 (95% CI, 0.51–0.92), *p* = 0.013 in the upper groups, respectively. However, for cardiac death and SCD, the difference in prognosis was significant only when comparing the upper with the lowest group, with HRs of 0.41 (95% CI, 0.19–0.87), *p* = 0.021 and HR of 0.23 (95% CI, 0.05–0.96), *p* = 0.044, respectively.

Similar results were obtained for total mortality and cardiac mortality after censoring the first 3 months. The univariate and multivariable HRs for 25(OH)D groups for the complete follow-up and after censoring the first 3 months are presented in Tables 2 and 3, respectively.

**Table 2:** **The univariate and multivariable HRs (95% CI) for groups of 25(OH)D for the complete 2-year follow-up**.

	Univariate analysis	Multivariable analysis
	>70 vs. ≤40 nmol/L	>70 vs. ≤40 nmol/L
Total mortality	0.43 (0.28–0.66), *p* < 0.001	0.56 (0.34–0.92), *p* = 0.021
Cardiac death	0.33 (0.17–0.62), *p* = 0.001	0.41 (0.19–0.87), *p* = 0.021
SCD	0.16 (0.05–0.52), *p* = 0.002	0.23 (0.05–0.96), *p* = 0.044

	**Univariate analysis**	**Multivariable analysis**
	**40–≤70 vs. ≤40 nmol/L**	**40–≤70 vs. ≤ 40 nmol/L**

Total mortality	0.49 (0.38–0.64), *p* < 0.001	0.69 (0.51–0.92), *p* = 0.013
Cardiac death	0.57 (0.41–0.80), *p* = 0.001	0.83 (0.56–1.23), *p* = 0.35
SCD	0.51 (0.32–0.81), *p* = 0.005	0.23 (0.05–1.32), *p* = 0.32

*SCD, sudden cardiac death*.

**Table 3:** **The univariate and multivariable HRs (95% CI) for groups of 25(OH)D after censoring events in the first 3 months of follow-up**.

	Univariate analysis	Multivariable analysis
	>70 vs. ≤40 nmol/L	>70 vs. ≤40 nmol/L
Total mortality	0.33 (0.18–0.58), *p* < 0.000	0.38 (0.19–0.74), *p* = 0.005
Cardiac death	0.24 (0.09–0.60), *p* = 0.002	0.26 (0.08–0.85), *p* = 0.027
SCD	0.23 (0.07–0.78), *p* = 0.010	0.39 (0.09–1.71), *p* = 0.210

	**Univariate analysis**	**Multivariable analysis**
	**40–≤70 vs. ≤40 nmol/L**	**40–≤70 vs. ≤40 nmol/L**

Total mortality	0.48 (0.35–0.66), *p* < 0.000	0.69 (0.49–0.97), *p* = 0.033
Cardiac death	0.60 (0.39–0.91), *p* = 0.016	0.96 (0.60–1.55), *p* = 0.88
SCD	0.47 (0.27–0.84), *p* = 0.10	0.86 (0.45––1.62), *p* = 0.63

*SCD, sudden cardiac death*.

Adding 25(OH)D levels to the significant confounders improved the prognostic value of the Cox regression model, using the censored values for both all-cause mortality [χ^2^(2) = 9.723; *p* = 0.008] and cardiac death [χ^2^(2) = 7.647; *p* = 0.022].

### Prognosis Estimates Based on Set Cut-Off Values of Vitamin D

In the multivariable analysis, there was a statistically significant decrease in all-cause mortality in the population with 25(OH)D level above 40 nmol/L as compared to those with a level below or equal to 40 nmol/L, with a HR of 0.66 (95% CI, 0.50–0.88), *p* = 0.004, whereas no significant difference was found in relation to cardiac and SCD. Furthermore, these differences were maintained when assessing the total population according to gender.

In the multivariate analysis, we also noted a statistically significant decrease in cardiac death in patients with 25(OH)D levels above 70 nmol/L as compared to those with levels equal to or below 70 nmol/L, with a HR of 0.46 (95% CI, 0.22 – 0.94), *p* = 0.034, whereas no significant difference was found in relation to total mortality and SCD. Furthermore, no significant differences were found when assessing the total population according to gender.

### Blood Donors From Argentina and Norway

Mean age of the total blood donor group (*n* = 190) was 46.0 ± 10.3 years. There were 96 females with a mean age of 46.1 ± 10.4 years, and a 25(OH)D level of 59.7 ± 21.5 nmol/L. The mean age of the male population (*n* = 94) was 46.0 ± 10.3 years, and this gender presented with a 25(OH)D level of 55.3 ± 18.2 nmol/L. No gender differences in 25(OH)D levels were found in this normal population. Values for the separate countries are presented in Table 4.

**Table 4:** **The baseline characteristics according to 25(OH)D concentrations in blood donors according to the country**.

Norway	25(OH)D (nmol/L)	Argentina	25(OH)D (nmol/L)	Total population	25(OH)D (nmol/L)
Total (*n* = 86)	62.4 ± 22.1	Total (*n* = 104)	53.5 ± 17.2	Total (*n* = 190)	57.5 ± 20.0
Age < 50 (*n* = 50)	59.4 ± 21.6	Age < 50 (*n* = 53)	51.4 ± 17.3	Age < 50 (*n* = 103)	55.3 ± 19.8
Age ≥ 50 (*n* = 36)	66.5 ± 22.5	Age ≥ 50 (*n* = 49)	55.2 ± 17.1	Age ≥ 50 (*n* = 85)	60.0 ± 20.2
Females (*n* = 43)	66.1 ± 22.6	Females (*n* = 53)	54.5 ± 19.3	Females (*n* = 96)	59.7 ± 21.5
Males (*n* = 43)	58.6 ± 21.2	Males (*n* = 51)	52.5 ± 14.9	Males (*n* = 94)	55.3 ± 18.2

## Discussion

As optimal and exact cut-off levels of vitamin D are still under debate, we have addressed this issue in the present analysis and studied the prognostic value of vitamin D cut-off values of 40 and 70 nmol/L, respectively, in a pooled ACS suspected patient population from inland Argentina and coastal-Norway, applying univariate and multivariate analyses. Furthermore, survival was compared for patients with vitamin D levels ≤40 nmol/L, >40 to ≤70 nmol/L, and >70 nmol/L, respectively. The proportion of total-, cardiac- and SCD decreased significantly with increasing vitamin D levels in the univariate analysis. This pattern was maintained for total death in the multivariable analysis, whereas cardiac death and SCD differed only when comparing the highest to the lowest group. Adding 25(OH)D levels to the significant confounders improved the prognostic value of the Cox regression model.

We chose a cut-off level of 40 nmol/L for vitamin D, based on a report by Ross et al (12), claiming that a vitamin D concentration above this level will meet the needs of 50% of the population. As demonstrated previously (10), patients in the lower vitamin D group had a significant lower survival rate than those with levels above 40 nmol/L. These differences were maintained when assessing the total population according to gender. In our population, 28% of the patients had vitamin D levels below or equal to 40 nmol/L. Patients in the lower level vitamin D group were older, had higher BMI, and were sicker as judged by a higher rate of heart insufficiency and diabetes as compared to patients in the higher vitamin D group. As suggested by the US Endocrine Society, levels below 50 nmol/L are defined as deficiency/insufficiency and levels above 75 nmol/L are regarded as sufficient (13). Accordingly, we chose a second cut-off level of 70 nmol/L and above this level we demonstrated a statistically significant reduced HR of cardiac death.

It has previously been reported that also high concentrations of 25(OH)D are associated with elevated risks of overall mortality (3, 14), but this relationship was not noted in the present study.

Vitamin D measurements were performed in blood samples obtained at admission, as close as possible to the index event, decreasing the possibility of a fall of vitamin D during hospitalization and reconvalescence. Mean vitamin D levels in our study patient population were similar to that of a healthy blood donor population, suggesting that low vitamin D levels are not necessarily secondary to patients’ health status. The similarity in vitamin D status between patients and healthy individuals may support the utility of vitamin D as a primary predictor of future outcome in our ACS patient population.

With the applied cut-off levels of 40 and 70 nmol/L, the sensitivity and specificity favors the use of the former cut-off level, demonstrating a sensitivity of 0.435 and specificity of 0.744, whereas a cut-off level of 70 nmol/L is associated with a sensitivity of 0.898 and a low specificity of 0.150.

Strengths consist of an unselected chest pain population with suspected ACS and a relatively short follow-up time of 2 years.

Limitations have previously been described (9, 10) and consist mainly of one set of blood samples at admission and no measurements of parathyroid hormone. As this is an observational study, unknown and residual confounders may have been missed. The “parent” studies RACS and ARRA-RACS were performed in two countries in different hemispheres with different sun exposure and dietary habits. Lifestyle confounders, including physical activity, exposure to sunlight, diet, and alcohol consumption are difficult to measure and could potentially influence the results. Furthermore, reverse causation is inherent in an observational study; thus, vitamin D concentrations could simply be an indicator of chronic disease or a marker of a lifestyle. Although blood samples were drawn in each country, vitamin D analysis was performed by one biochemist at the Department of Medical Biochemistry at Stavanger University Hospital.

In conclusion, we noted an increased rate of total mortality with a level of vitamin D below 40 nmol/L and increased cardiac mortality was found to be related to vitamin D levels below 70 nmol/L. The results might have practical implications in terms of prophylactic vitamin D treatment in subjects at high risk of coronary heart disease, but we need randomized intervention studies to assess whether vitamin D supplementation reduces mortality and CVDs.

## Author Contributions

PN: contributed to study design, data collection, vitamin D analysis, interpretation of results, and preparation of the manuscript. RF: contributed to study design, data collection, clinical follow-up, interpretation of results and preparation of the manuscript. STN: contributed to study design, interpretation of results, and preparation of the manuscript. VP: contributed to study design, data collection, clinical follow-up, interpretation of results, and preparation of the manuscript. TB-A: contributed to study design, data collection, clinical follow-up, interpretation of results, and preparation of the manuscript. HG: contributed to study design, data collection, interpretation of results, and preparation of the manuscript. HS: contributed to data collection, interpretation of results, and preparation of the manuscript. DN: conceived the idea of the study, supervised the study, including interpretation of results, and preparation of the manuscript. All authors have read and approved the final manuscript.

## Conflict of Interest Statement

The authors declare that the research was conducted in the absence of any commercial or financial relationships that could be construed as a potential conflict of interest.
